# Supplementary material for: Complex‐centric proteome profiling by SEC‐SWATH‐MS
Source: Mol Syst Biol. 2019 Jan 14;15(1):e8438. doi: 10.15252/msb.20188438 (PMC6346213; doi:10.15252/msb.20188438)
Supplement: Supplementary file 6 — Dataset EV5 [file MSB-15-e8438-s006.zip › feature_plots_corum/2721-1.pdf]

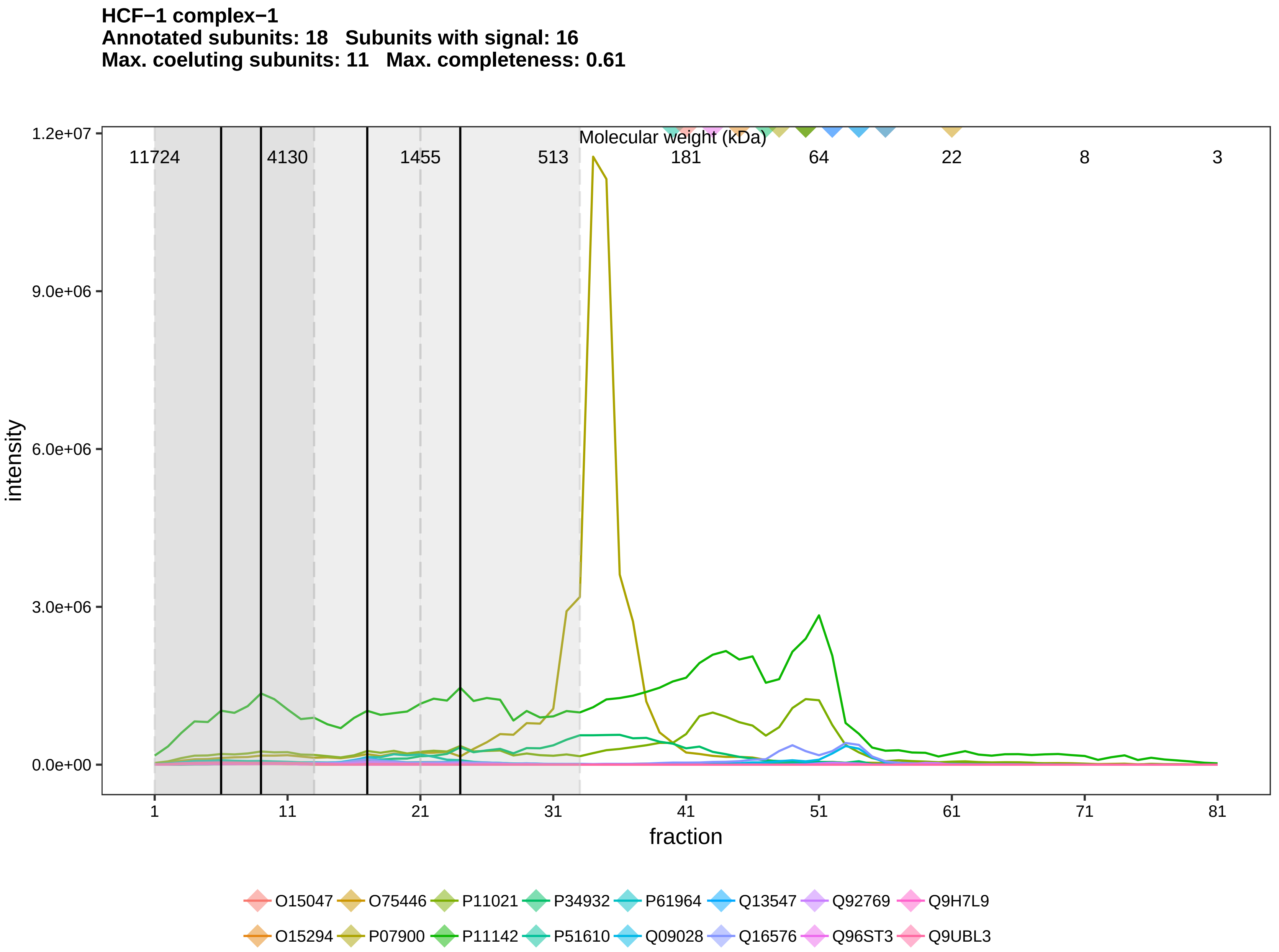

HCF−1 complex−1
Annotated subunits: 18 Subunits with signal: 16
Max. coeluting subunits: 11 Max. completeness: 0.61
Molecular weight (kDa)
11724
4130
1455
513
181
64
22
8
3
1.2e+07
9.0e+06
6.0e+06
3.0e+06
0.0e+00
intensity
1
11
21
31
41
51
61
71
81
fraction
O15047
O75446
P11021
P34932
P61964
Q13547
Q92769
Q9H7L9
O15294
P07900
P11142
P51610
Q09028
Q16576
Q96ST3
Q9UBL3
